# *PUM1* and *RNase P* genes as potential cell‐free DNA markers in breast cancer

**DOI:** 10.1002/jcla.23720

**Published:** 2021-02-01

**Authors:** Alexis Murillo Carrasco, Oscar Acosta, Jaime Ponce, José Cotrina, Alfredo Aguilar, Jhajaira Araujo, Pamela Rebaza, Joseph A. Pinto, Ricardo Fujita, José Buleje

**Affiliations:** ^1^ Facultad de Medicina Humana Centro de Investigación de Genética y Biología Molecular Instituto de Investigación Universidad de San Martín de Porres Lima Perú; ^2^ Facultad de Farmacia y Bioquímica Universidad Nacional Mayor de San Marcos Lima Perú; ^3^ Oncosalud–AUNA Unidad de la Mama Lima Perú; ^4^ Departamento de Cirugía de Mamas Instituto Nacional de Enfermedades Neoplásicas–INEN Lima Perú; ^5^ Oncosalud–AUNA Unidad de Investigación Básica y Traslacional Lima Perú

**Keywords:** breast cancer, cell‐free DNA, digital PCR, liquid biopsy, ultrasensitive detection method

## Abstract

**Background:**

Cell‐free DNA (cfDNA) is used in clinical research to identify biomarkers for diagnosis of and follow‐up on cancer. Here, we propose a fast and innovative approach using traditional housekeeping genes as cfDNA targets in a copy number analysis. We focus on the application of highly sensitive technology such as digital PCR (dPCR) to differentiate breast cancer (BC) patients and controls by quantifying regions of *PUM1* and *RPPH1* (*RNase P*) in plasma samples.

**Methods:**

We conducted a case‐control study with 82 BC patients and 82 healthy women. cfDNA was isolated from plasma using magnetic beads and quantified by spectrophotometry to estimate total cfDNA. Then, both PUM1 and RPPH1 genes were specifically quantified by dPCR. Data analysis was calibrated using a reference genomic DNA in different concentrations.

**Results:**

We found *RNase P* and *PUM1* values were correlated in the patient group (intraclass correlation coefficient [ICC] = 0.842), but they did not have any correlation in healthy women (ICC = 0.519). In dPCR quantification, *PUM1* showed the capacity to distinguish early‐stage patients and controls with good specificity (98.67%) and sensitivity (100%). Conversely, *RNase P* had lower cfDNA levels in triple‐negative BC patients than luminal subtypes (*p* < 0.025 for both), confirming their utility for patient classification.

**Conclusion:**

We propose the *PUM1* gene as a cfDNA marker for early diagnosis of BC and *RNase P* as a cfDNA marker related to hormonal status and subtype classification in BC. Further studies with larger sample sizes are warranted.

## Graphical abstract

Liquid biopsies could offer interesting data of the neoplastic process in one patient. Then, this study analyzed cell‐free DNA (cfDNA) from human plasma to assess two housekeeping genes as DNA markers for breast cancer in a chip‐based platform. Our results indicate cfDNA regions of *PUM1* and *RNAse P* genes as able to distinguish between early‐stage breast cancer patients and healthy women in a Peruvian cohort.

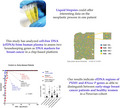

## 1 INTRODUCTION

Breast cancer (BC) is the most lethal malignancy in women around the globe,^1^ and this is true regardless of the socioeconomic features of each country.^2^ To date, mammogram‐guided biopsies continue to be the gold standard for BC diagnosis despite their limited sensitivity (80%), which eventually complicates the identification of tumors in dense breast tissue.^3^ As a result, epidemiological studies have proposed early diagnosis of BC as a way to increase 5‐year survival rates for these patients, and more recently, liquid biopsies have seemed to support this objective by the constant monitoring of suspected high‐risk individuals.^2^, ^4^, ^5^

This study focused on cell‐free DNA (cfDNA), a marker of liquid biopsies that represents fragmented nuclear DNA that is released during the cell death process in our cells, which could potentially be isolated from plasma or other bodily fluids.^6^ An overpopulation of any cell type, such as cancer, drives high levels of cfDNA seen in a liquid biopsy (eg, blood sample). Since their first description, cfDNA has been associated with the characterization of cancer patients,^7^ and it has been recently reported to be elevated in samples of various cancers, including gastric,^8^ bladder,^9^ lung,^10^ and breast.^11^

These previous studies have proven the potential of cfDNA markers for the early diagnosis and prognosis of cancer; however, their origin and nature expose some limitations when studying them, such as the lack of a gold standard technique to analyze cfDNA or common markers for different cancer types. Regarding the low concentration of cfDNA in plasma,^12^ this study proposed to analyze samples with a highly sensitive technology such as digital PCR (dPCR), because this technique allows us to obtain an absolute copy number of cfDNA that is different from previously used techniques for evaluating cfDNA.^13^

Theoretically, different regions of nuclear DNA could be released into human plasma, but their half‐lives are variable and reduced due to the presence of nucleases, which affect the concentration of these regions in cfDNA. However, the selection of the best marker for each cancer type depends on their oncogenic potential, suggesting a hypothesis about the preferential segregation of certain regions.^14^, ^15^, ^16^ This selection could prefer regions with a stable expression (housekeeping genes), as indicated by the experiments of Huang et al^17^ and Zhong et al^18^ targeting β‐globin or glyceraldehyde 3‐phosphate dehydrogenase (GAPDH) genes in BC patients and healthy women.

In our study, we tested the potential of two genomic regions to be cfDNA markers: *RPPH1* (*RNase P*) and *PUM1*. These genes are traditionally used as housekeeping genes in several expression studies and show preserved sequences.^19^, ^20^, ^21^, ^22^ *PUM1*, in particular, demonstrated the most consistent expression in a very comprehensive analysis of The Cancer Genome Atlas (TCGA) RNA‐seq data.^23^

Briefly, this study proposes a new approach to testing the two traditional housekeeping genes *RNase P* and *PUM1* as cfDNA biomarkers in plasma to evaluate their characteristics for early diagnosis and prognosis of BC. Our protocol involves a less invasive method for sample collection and a fast high‐performance technology for molecular analysis, without the need to analyze mutations.

## 2 METHODS

### 2.1 Samples

We conducted a prospective case‐control study with 82 BC patients representing 1.2% of all new cases of BC reported in 2018 in Peru.^24^ We also included 82 controls (women who underwent BC screening with negative results).

For the disease group, we enrolled patients recently diagnosed with BC (with histological confirmation) at both private and public cancer centers in Lima (Oncosalud–AUNA and Instituto Nacional de Enfermedades Neoplásicas–INEN). For the control group, we enrolled women without benign or malignant breast lesions as determined by mammogram, ultrasonography, and clinical examination at Oncosalud–AUNA and Universidad de San Martín de Porres. All participants signed an informed consent document before blood collection, according to the protocol IRB00003251‐FWA0015320 (Universidad de San Martín de Porres/Clínica “Cada Mujer”). Samples and patients' information were coded to maintain the anonymity of participants.

### 2.2 Clinical variables

For the BC patients, we collected clinical information related to age at diagnosis, hormonal status, clinical stage, and histological type. Other clinical variables related to non‐oncogenic cfDNA production, such as lymphocyte and glucose levels (Table S1), were also taken.

### 2.3 Plasma separation and cfDNA isolation

Blood samples were centrifuged at 4°C for 10 minutes at 1200 *g*. The plasma was collected, centrifuged at 4 C for 10 minutes at 16,000 g, and stored in 2.0 ml cryogenic tubes at −80°C. cfDNA was then extracted using the MagMAX Cell‐Free DNA Isolation Kit (Applied Biosystems) following the manufacturer's instructions. Briefly, 12 μl of magnetic DNA‐affinity beads and 1 ml of binding buffer were used per 0.8 ml of plasma sample. The tube content was mixed for 10 minutes and placed in a magnetic stand for 5 minutes. After magnetic separation, the supernatant was discarded. The tubes were then removed from the magnet, and 1 ml of wash buffer was added, mixed, and transferred to new tubes before being placed back into the magnetic separation rack. After two washes with 80% ethanol, the beads were allowed to dry with the lid of the tube open for 6 minutes. Finally, 40 μl of elution buffer was added directly to the beads and mixed for 5 minutes before placing the sample back in the magnetic rack for 2 minutes. Eluted DNA was recovered from each tube, leaving the beads attached to the walls, and cfDNA tubes were frozen at −20°C until their use.

### 2.4 Quantification of total cfDNA

Total cfDNA from the samples was quantified using spectrophotometry. For this, 1.5 μl of each sample was put in the Nanodrop spectrophotometer platform (Thermo Fisher Scientific), using elution buffer as blank.

### 2.5 Quantification by digital PCR

cfDNA levels in BC patients and controls were evaluated with detection assays (TaqMan Copy Number Assay) for both *PUM1* (amplicon length: 77 bp, assay Hs00956027_cn, catalog number 4400291; Thermo Fisher Scientific) and *RNase P* (amplicon length: 87 bp, specifically *RPPH1*, catalog number 4403326; Thermo Fisher Scientific). These two assays are characterized by their small amplicon size, location in exonic regions, and having no described genomic variants within them. Each sample was amplified using 1.5 μl of cfDNA, 0.75 μl of each probe (PUM1 and *RNase P*), and 7.5 μl of QuantStudio 3D Digital PCR Master Mix v2 (catalog number A26359; Thermo Fisher Scientific) up to a final volume of 15 μl. All amplification mixes were dispensed into QuantStudio3D chips for dPCR (Thermo Fisher Scientific). To prevent evaporation, mineral oil was added, and the chips were sealed and placed in a ProFlex thermocycler (Applied Biosystems). Chip content was amplified using the following program: initial denaturation at 96°C for 10 minutes, followed by 44 cycles of hybridization and extension at 60°C for 2 minutes, denaturation at 98°C for 30 seconds, and a final extension at 60°C for 2 minutes. Immediately after amplification, the chips were stored at 10°C until the reading step. The latter was performed in the QuantStudio3D reader (Thermo Fisher Scientific), and results were analyzed with the QuantStudio 3D Analysis Suite Software (Thermo Fisher Scientific). Finally, the units of all cfDNA levels were transformed from copies per sample microliter (reported by software) to copies per plasma milliliter (copies/ml of plasma).

### 2.6 Analytical assessment

A reference sample of human genomic DNA was used to verify the amplification of the PUM1 and *RNase P* assays through dPCR. We prepared dPCR chips (QuantStudio3D) with different DNA concentrations (50 ng/µl, 25 ng/µl, and 12.5 ng/µl) to assess thresholds for amplification. We aimed to distinguish the proper detection of a sample from the artifacts derived from chip saturation or nonspecific amplification. All chips were amplified following the manufacturer's instructions. The results obtained were registered and used to calibrate the amplification thresholds.

### 2.7 Image analysis and statistical tests

The graphs and values were analyzed using QuantStudio3D Analysis Suite v.3.1.3 Cloud Software (Thermo Fisher Scientific). Only fluorescent dots with more than 40% quality (self‐determined by software) were selected for analysis to avoid artifacts. Statistical comparisons were performed in Prism 7 software (GraphPad Software Inc.) using the Mann‐Whitney test with Bonferroni correction for *p*‐Values and receiver operating curve (ROC) analysis.

## 3 RESULTS

### 3.1 Breast cancer clinical data

The mean age of participants was 55.61 years (standard deviation [SD], ±13.15) in controls (*N* = 82) and 53.88 (SD, ±11.93) in BC patients (*N* = 82, *p* = 0.5349). Regarding the clinical stage in patients with BC, 45% were in early stages (18% in stage I and 26% in stage II). The rest were in advanced stages (47% in stage III and 8% in stage IV). Ductal histology was most frequent (77%), followed by lobular tumors (13%). Tumors expressing the estrogen receptor, progesterone receptor, and human epidermal growth factor receptor (HER2) were 68%, 51%, and 27%, respectively (Table 1). Further clinical information, including lymphocyte and glucose levels, is presented in Table S1.

**TABLE 1:** Sample distribution by clinical characteristics

Clinical features	Years
Age	53.97 ± 11.9
**Stages**	**Frequency**
Early	0.45
I	0.19
II	0.26
Advanced	0.55
III	0.47
IV	0.08
**Histological Type**	**Frequency**
Ductal	0.77
Lobular	0.13
Mixed	0.05
Others	0.05
**ER Status**	**Frequency**
Positive	0.68
Negative	0.32
**PR Status**	**Frequency**
Positive	0.51
Negative	0.49
**HER2 Status**	**Frequency**
Positive	0.27
Negative	0.73

Note

The values are shown as mean ± standard deviation (SD) or relative frequency.

Abbreviations: ER, Estrogen receptor; HER2, human epidermal growth factor receptor 2; PR, progesterone receptor.

### 3.2 Determination of fluorescence thresholds

**FIGURE 1:**
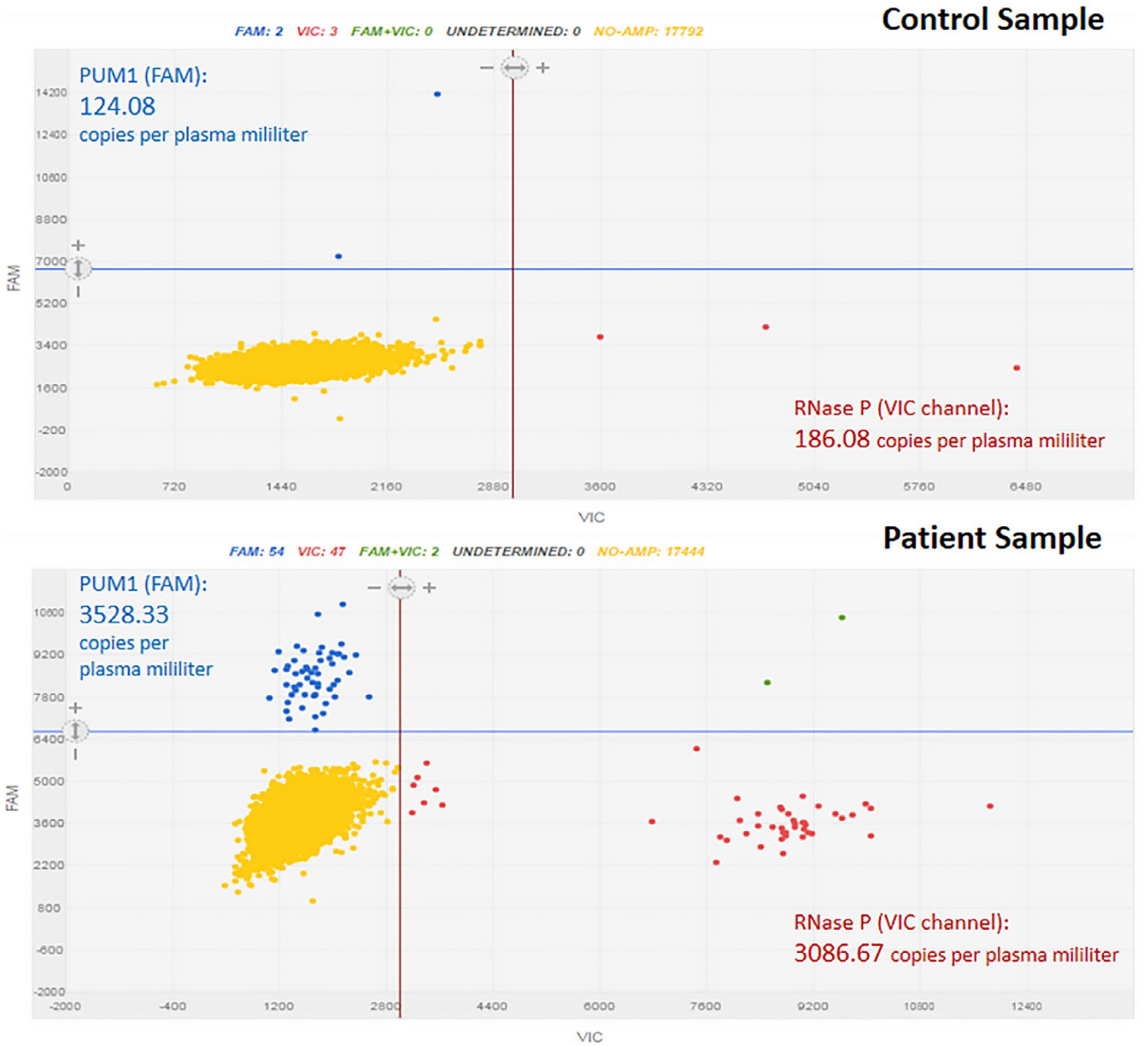
Digital PCR (dPCR) supports a high‐sensitivity platform to determine absolute DNA quantification. An analytical control was run to assess the capacity of dPCR to distinguish differences between control and patient samples. Shown are two samples (control and patient), where blue and red dots are positive wells for *PUM1* and *RNase P*, respectively, green dots are positive wells for both markers, and yellow dots represent neither amplification. Estimated concentrations of cfDNA (copies/ml of plasma) are indicated for each case

Our study considered that samples with less than 50 ng/µl of cfDNA were satisfactory for loading in dPCR chips. This consideration allowed for the use of plasma samples despite their high protein content, which could cause interference in the results. To avoid these interferences, we established fluorescence thresholds for both the VIC and FAM dye channels: 6600 relative fluorescence units (RFU) on the FAM dye channel (PUM1 quantification, Y‐axis) and 3000 RFU on the VIC dye channel (*RNase P* quantification, X‐axis). These custom parameters allowed for the identification of differences between the patient and control groups (Figure 1).

### 3.3 Patients showed greater correlation of *PUM1* and *RNase P* than controls

We did not find a normal distribution of cfDNA levels in all individuals enrolled in the study, according to the Kolmogorov–Smirnov test (*p* < 0.01, indicating nonparametric tests for our data). Afterward, we evidenced a miscorrelation among the total and specific quantifications of cfDNA. Total quantification (by spectrophotometry) reported a range of 8.3–75 ng/ml of plasma for all samples, while dPCR quantification (for *PUM1* and *RNase P* genes) described an equivalent of 0–48.2 ng/ml of plasma. These results were consistent with a pilot study where dPCR (for the same genes) and Qubit fluorometer quantification showed different values for the same sample group (*R* ^2^ = 0.2531 for *PUM1* and 0.2781 for *RNase P*; Figure S1). Interestingly, both *RNase P* and *PUM1* seemed to have been released equally in the patient group, showing an intraclass correlation coefficient (ICC) equal to 0.842. However, in the control group, the same markers showed a weak correlation (ICC = 0.519, Figure S1). These findings suggest that some genomic regions, such as *RNase P*, have more consistent release into the bloodstream than *PUM1*, even in healthy women.

### 3.4 *RNase P* had lower cfDNA levels in triple‐negative BC patients than luminal subtypes

**FIGURE 2:**
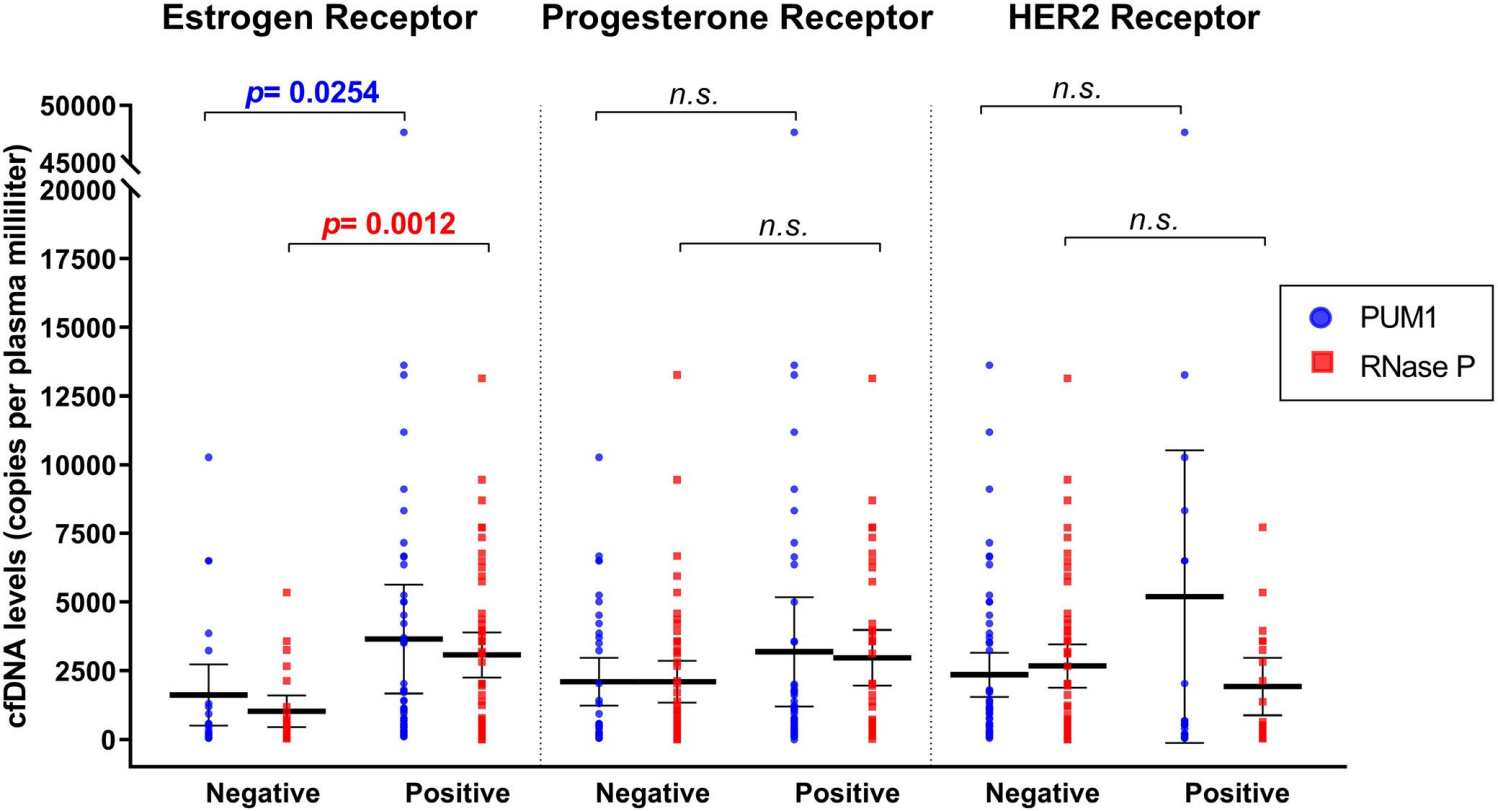
Estrogen‐positive patients present higher levels of *RNase P*. All samples were classified according to immunohistochemical conditions, and cfDNA levels for *PUM1* and *RNase P*are represented in blue and red dots, respectively. A small rise of levels in the estrogen‐ or progesterone‐positive groups was shown; however, the results for the HER2 receptor might be relative to the tested marker. The *p*‐values were calculated with the Mann‐Whitney test with Bonferroni correction. n.s.: non‐significant

With regard to immunohistochemical (IHC) profiles, patients expressing the estrogen receptor presented higher cfDNA levels of *PUM1* (*p* = 0.0254) and *RNase P* (*p* = 0.0012). In contrast, we did not find differences in the expression of the progesterone receptor (*p* = 0.3036 for *PUM1* and *p* = 0.0887 for *RNase P*) or for HER2 overexpression (*p* = 0.6967 for *PUM1* and *p* = 0.2817 for *RNase P*) (Figure 2). Consequently, we also found fewer copies of *RNase P* in triple‐negative patients (mean ± SD = 649.3 ± 709.7 copies/ml of plasma) than Luminal A (mean ± SD = 3132 ± 3258 copies/ml of plasma; *p* = 0.0221) or Luminal B (mean ± SD = 2907 ± 2555 copies/ml of plasma; *p* = 0.0145) subtypes.

### 3.5 *PUM1* showed greater specificity and sensitivity for classifying early‐stage BC patients

**FIGURE 3:**
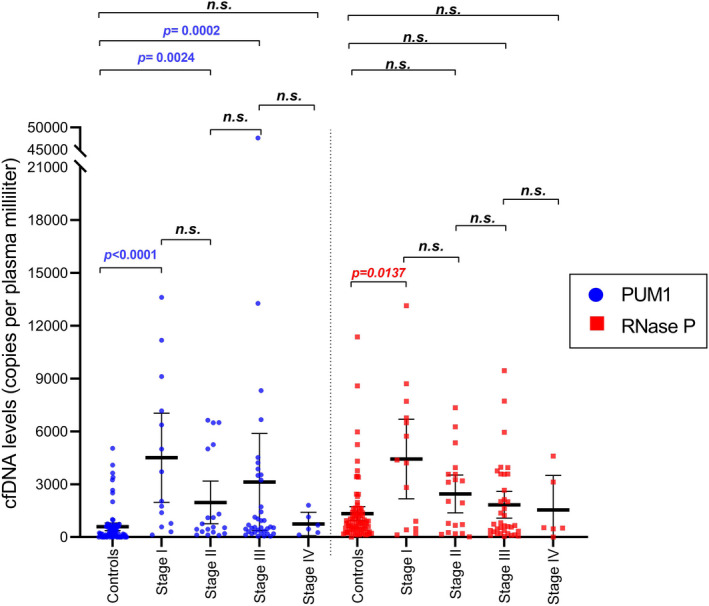
*PUM1* allows for distinguishing among patient and control groups. cfDNA was measured through digital PCR for *PUM1* and *RNase P*, and all data were plotted using a scatter dot graph for healthy women and each breast cancer stage. The *p*‐values were calculated with the Mann‐Whitney test with Bonferroni correction. n.s.: non‐significant

**FIGURE 4:**
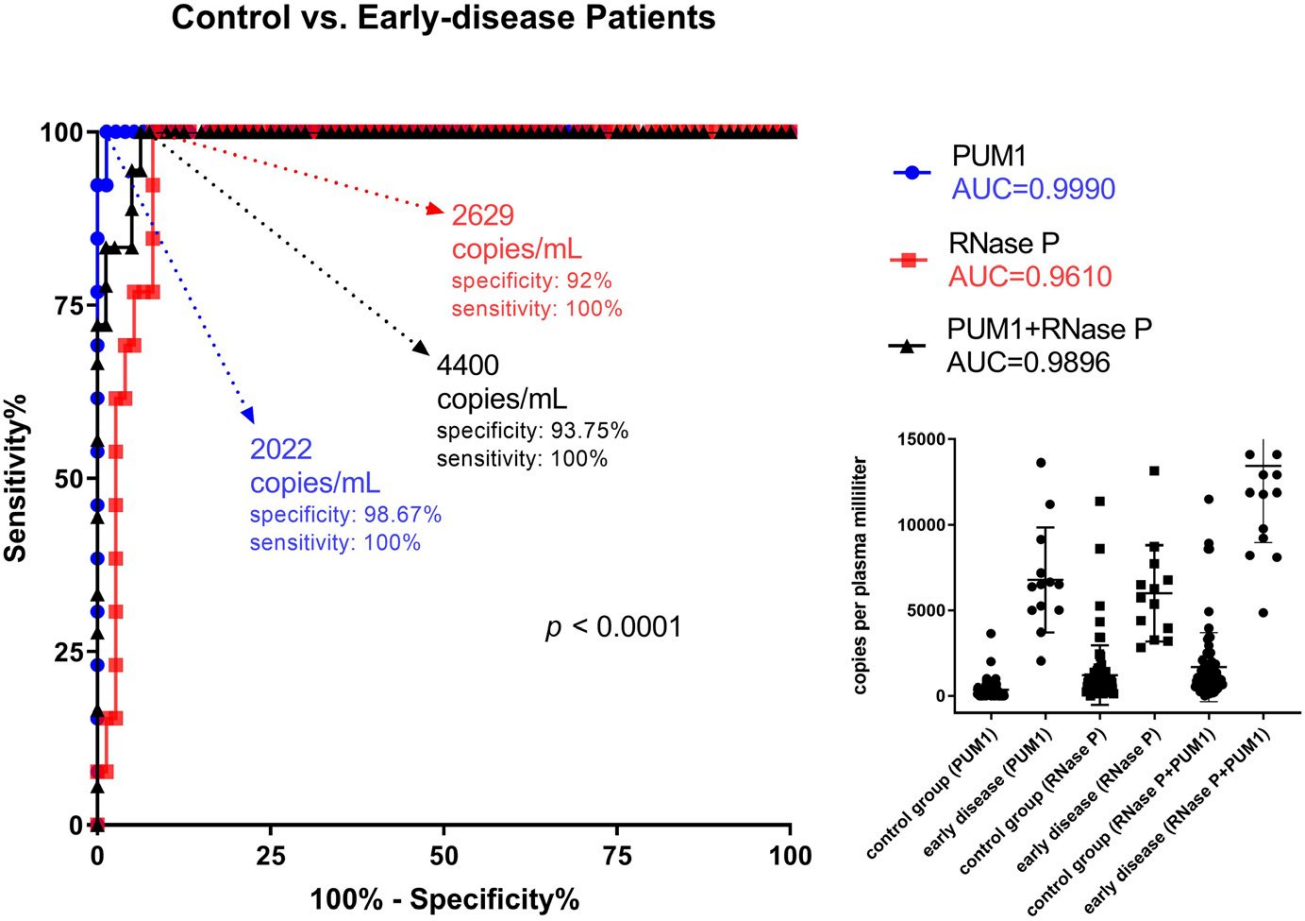
*PUM1* shows better conditions for the early determination of breast cancer. ROC analysis for *PUM1* and *RNase P* assays was performed among the control group and early‐stage patients. Both had significant results, but *PUM1* featured a high specificity and lower cutoff value, according to the extended results shown in the dot plot.

In a broad comparison among healthy women and each clinical stage of BC patients, *RNase P* levels were different in the controls and BC patients in stage I (*p* < 0.01), whereas *PUM1* levels were lower in controls than in BC patients in stages I*–*III (Figure 3). Next, our study tested the ability of *RNase P* and *PUM1* to indicate early‐stage BC patients through the ROC. We established the best cut‐off point based on both specificity (spec) and sensitivity (sens) analyses, resulting in 2002 copies/ml for *PUM1* (spec = 98.67%; sens = 100%), 2629 copies/ml for *RNase P* (spec = 92%; sens = 100%), and 4400 copies/ml for the combination of both markers (spec = 93.75%; sens = 100%). These cutoff points generated areas under the curve (AUC) of 0.999 for PUM1, 0.961 for *RNase P*, and 0.9896 for the combination of both markers (Figure 4). This result indicates that *PUM1* and *RNase P* are good biomarkers for differentiating BC patients and healthy women, despite *PUM1* showing better performance with a lower threshold (expressed in copies per ml of plasma).

## 4 DISCUSSION

The use of cfDNA in liquid biopsy has gained relevance for precision medicine in recent years, mainly due to its versatility in different approaches to studying cancer. For instance, cfDNA could be analyzed with the aim of searching for specific mutations in EGFR/ALK genes,^25^ identifying driver mutations in a group of cancer patients,^26^ or broadly exploring and analyzing methylated cfDNA in BC patients.^27^ Our study proposes a practical and innovative method of analyzing cfDNA, focusing on only the absolute copy number of specific DNA regions in human plasma, without the need to analyze mutations.

Our hypothesis suggests the presence of a higher number of copies of cfDNA regions in early‐stage patients than in controls, and we propose the use of cfDNA analysis in a fast, specific, and highly sensitive way through dPCR. Our method is fast because after standardization, it would take only approximately 7 hours from blood collection to quantification of the absolute number of cfDNA copies. There are several reports describing differences in cfDNA concentrations among patients and control individuals, and each used different protocols. These protocols diverged in the isolation method of either centrifugation^28^ or column purification^29^, ^30^, ^31^ and in the quantification method of either spectrophotometry^31^ or PCR‐based techniques.^30^ Consequently, we have various and relative units of reported data regarding cfDNA, which complicates the comparison of methods and the establishment of a gold standard. For that reason, we propose analysis with a technology expressing absolute quantification values.

In BC, Huang et al^17^ demonstrated that malignant breast lesions produced higher concentrations of DNA in serum (median 65 ng/ml) than both benign lesions (median 22 ng/ml) and healthy breasts (median 13 ng/ml). These statistically different concentrations (*p* < 0.05) were obtained when using silica columns and real‐time PCR for cfDNA extraction and quantification, respectively.^17^ More recently, Baselga et al^11^ used whole genome sequencing (WGS) to study genetic mutations, copy number variations, and methylation changes in a large cohort (almost 900 women), spearheading the use of cfDNA as a tool in liquid biopsy for an early diagnosis of BC.

cfDNA represents a very low percentage of total DNA present in the blood. In a pilot study, we estimated the mean value of cfDNA in the control group to be 10–100 ng/ml, representing 0.1% of the total amount of DNA in the blood. In this study, we did not find a correlation of the total quantification of cfDNA (determined by spectrophotometry) and the quantification of specific regions (*PUM1* and *RNase P*) by dPCR (Figure S1). These data open new possibilities for studying specific regions in cfDNA; however, it is probable that different results will be obtained when different markers are evaluated. The information in the literature about cfDNA indicates that it is fragmented in a cell equally regardless of the process and could then be digested by nucleases or even taken up by other cells in the bloodstream, driving the clearance of cfDNA.^32^ Previous studies demonstrated that long non‐coding regions of *RPPH1* act as tumor promoters and play an important role in advancing tumorigenesis by targeting miR‐122. They may also serve as a novel and potential prognostic target in BC.^33^ Conversely, however, a study that used dPCR and bioinformatics methods to identify genic fusion of *PUM1* and *TRAF3*, which was associated with poor survival in bile duct cancer patients,^34^ suggested that *PUM1* is involved in the initial tumorigenic process. To prevent any bias due to the assay selected, we evaluated the size of all available probes for the *PUM1* and *RNase P* regions (77 and 87 bp, respectively), their localization (preferring exonic regions), and the absence of previously described genomic variants, which could produce variable results.

Several authors have described common factors influencing the levels of total cfDNA (eg, age, sex, hormonal status, number of lymphocytes in blood, glucose levels), even in healthy populations.^12^, ^28^, ^29^, ^30^, ^31^, ^35^ These factors may dismiss the simple quantification of total cfDNA to predict the oncogenic process; however, we propose the highly sensitive quantification of specific regions to improve this method. To discard biological factors interfering in our cfDNA quantification, we selected women of a similar age for both the control and patient groups (*p* = 0.5349). Neither group had high glucose levels, and only one patient showed lymphocytosis (lymph count >4000 cells per mm^3^) (Table S1).

In other studies, researchers compared risk factors with cfDNA levels,^11^ where Peruvian cohorts were interesting as study populations due to their ancestry admixture.^36^ Moreover, the samples included in this study came from INEN, the Peruvian national reference center for cancer treatment. Interestingly, Zavala et al^37^ reported that some risk factors for BC in women (smoking, age at menarche, and full‐term pregnancies) are only related to the place of birth and not to the tumor subtype or stage.

Our study found a significantly higher copy number for *PUM1* (*p* < 0.05) and *RNase P* (*p* < 0.01) markers in patients expressing estrogen receptor (Figure 2), similar to other comparisons among BC subtypes. For instance, luminal groups showed a higher number of *RNase P* copies than triple‐negative groups (*p* < 0.025 for each). A low concentration of cell‐free markers in triple‐negative BC (TNBC) has been described in independent experiments using a proteomic approach.^38^, ^39^ However, the risk factors for our patients could not interfere with their cfDNA levels, justifying a potential function of *RNase P* regions in plasma in the oncogenic process.

Additionally, our data found a high concentration of cfDNA for *PUM1* and *RNase P* regions in stage I patients and no expected variability of these results in other stages (II–IV). We can support this data by the specific characteristics surrounding each stage of the cancerous process ^17^, ^40^; therefore, different cfDNA markers could be preferentially expressed in each stage despite total cfDNA showing a consistent increase (*p* < 0.05). Our study suggests that *PUM1 *has better diagnostic performance potential than *RNase P* or a combination of both markers for the discrimination of healthy women (control group) and early‐stage BC patients (AUC = 0.999, Figure 4). These differences are explained by the constant values of *PUM1* seen in the control group, highlighting this marker as less susceptible to common fluctuations of cfDNA content. This condition is essential for indicating a liquid biopsy marker because many putative markers exist for cfDNA (including SNVs, microsatellite instability, and loss of heterozygosity). However, only a small group of them would be associated with diseases such as BC.^41^ Other initiatives in the early diagnosis of BC report different markers, such as free miRNA,^42^, ^43^ exosomal miRNA,^44^ and proteins.^38^, ^45^, ^46^ Unfortunately, these markers for BC are not related, complicating the understanding of their participation in the tumor process. Here, we selected the *PUM1* gene because it has been shown in basic and clinical research to be an excellent housekeeping gene,^21^, ^22^, ^23^ and it was associated with poor prognosis for bile duct cancer through the formation of a genic fusion.^34^ Thus, our results reinforce a new application of this gene as a marker for cancer and suggest that future studies aim at the function of *PUM1* as cfDNA in cancer.

Using the *PUM1* gene, we established a cut‐off point of 2002 copies/ml of plasma to distinguish the control group and early‐stage patients, and this value fits with the broad spectrum (300–27,000 copies/ml of plasma) reported by other studies.^8^, ^47^, ^48^, ^49^ We also used the cut‐off point for PUM1 to perform the ROC curve analysis, and we obtained a very high specificity (98.67%) and sensitivity (100%) with an AUC equal to 0.99. These values are also comparable with other markers in plasma and other liquid biopsy sources, even in larger populations.^40^, ^43^, ^46^, ^50^ For instance, Gonzalez et al. described the protein RANTES in plasma as a diagnosis marker in 125 BC patients, with a range of AUC values (0.70–0.99), depending on the subtype.^46^ Another study testing 100 BC patients with miR‐30a in plasma showed a specificity of 65.6% and a sensitivity of 74%,^43^ compared with the sensitivity values of traditional biochemical markers such as CEA (12%) and CA153 (14%). More comprehensive cancer studies using all circulating tumor DNA (ctDNA) regions have shown a range of sensitivity of 59–98%, depending on the type of cancer and the subsequent rate of detection of mutations.^40^, ^50^

In brief, the implementation of different analytes as early diagnosis markers requires different conditions for each of them. However, nucleotide‐based markers appear to have better features than biochemical markers for the early diagnosis of BC.^43^ Consequently, an ideal marker for early diagnosis would be related to sensitivity, and this usually depends on their levels in healthy people; however, we found a broad range of normal levels of cfDNA across several studies.^8^, ^47^, ^48^, ^49^ This range is related to the methods used for plasma separation, DNA isolation, and DNA quantification, generating a great discussion about contaminants and highly sensitive detection methods. Today, we also need to establish a comparison point among the several units available for studies in this field (eg, copies/ml, ng/µl, genome equivalents); nonetheless, a large project using different technologies would be necessary to bridge this gap.

Here, we present both *RNase P* and *PUM1* as cfDNA markers with the potential to classify early‐stage BC patients and distinguish them from controls. These findings should support the improvement of the quality of life in early‐diagnosed patients.^4^ However, we still need to replicate our study in a larger population to eliminate the risk of misdiagnosed individuals.

Tests based on liquid biopsies should be compared with traditional screening tests like mammograms for BC. Unfortunately, mammograms are criticized for their high rates of overdiagnosis and overtreatment, which are related to poor survival due to adverse effects of drugs in patients.^51^ Finally, we strongly believe that *PUM1* and *RNase P* as cfDNA markers in plasma could provide a valuable tool for BC screening in conjunction with mammograms to obtain an improved selection of patient candidates for biopsy or closer follow‐up.

## 5 CONCLUSION

Due to high levels of usefulness and minimal invasiveness, tests based on liquid biopsies are needed to improve the quality of life for BC patients. Here, we reported on a new application of two traditional housekeeping genes as cfDNA markers in liquid biopsies. We propose the *PUM1* gene as a cfDNA marker for early diagnosis of BC and *RNase P* as a cfDNA marker related to hormonal status and subtype classification in BC.

## Supporting information

Supplementary Material
