# Supplementary material for: PUM1 and RNase P genes as potential cell‐free DNA markers in breast cancer
Source: J Clin Lab Anal. 2021 Feb 1;35(4):e23720. doi: 10.1002/jcla.23720 (PMC8059717; doi:10.1002/jcla.23720)
Supplement: Supplementary file 1 — Supplementary Material [file JCLA-35-e23720-s001.docx]

**Table S1.** Clinical Information from participant patients. All information was given by Oncosalud or INEN, NA: Not available.

| Code | Stage | Age at  diagnosis | Histology | ER | PR | HER2 | Lymphocyte Count (cells/mm3) | Blood  Group | Glucose  (mg/dL) |
| --- | --- | --- | --- | --- | --- | --- | --- | --- | --- |
| Fincyt-002-15 | III | 42 | ductal | Positive | Negative | Negative | 2927,40 | O+ | 93 |
| Fincyt-003-15 | I | 65 | lobular | Positive | Positive | Negative | 2285,40 | A+ | 98 |
| Fincyt-004-15 | I | 61 | lobular | Positive | Positive | Negative | 1684,80 | O- | 81 |
| Fincyt-005-15 | III | 46 | ductal | Positive | Positive | Positive | 1000,50 | O+ | 93 |
| Fincyt-006-15 | I | 72 | ductal | Positive | Positive | Negative | 720,00 | O+ | 88 |
| Fincyt-007-15 | II | 45 | ductal | Negative | Negative | Positive | 2023,10 | O+ | 89 |
| Fincyt-009-15 | II | 28 | ductal | Positive | Positive | Negative | 3773,60 | O+ | 94 |
| Fincyt-010-15 | I | 52 | mixed | Positive | Positive | Negative | 1872,00 | A+ | 94 |
| Fincyt-012-15 | I | 65 | ductal | Positive | Negative | Negative | 2167,30 | O+ | 94 |
| Fincyt-013-15 | I | 80 | ductal | Positive | Positive | Negative | 949,20 | O+ | 90 |
| Fincyt-014-15 | I | 42 | ductal | Positive | Positive | Negative | 2107,00 | A+ | 69 |
| Fincyt-015-15 | I | 68 | lobular | Positive | Positive | Negative | 2428,80 | O+ | 94 |
| Fincyt-016-15 | I | 60 | ductal | Positive | Negative | Positive | 1157,70 | O+ | 120 |
| Fincyt-017-15 | II | 70 | ductal | Negative | Negative | Negative | 1462,10 | A+ | 99 |
| Fincyt-018-16 | IV | 38 | ductal | Positive | Positive | Negative | 1278,00 | O+ | 80 |
| Fincyt-019-16 | I | 49 | lobular | Positive | Positive | Negative | 1537,20 | O+ | 92 |
| Fincyt-020-16 | I | 60 | mixed | Positive | Positive | Negative | 1413,60 | O+ | 108 |
| Fincyt-022-16 | II | 45 | ductal | Positive | Positive | Negative | 2192,92 | NA | NA |
| Fincyt-023-16 | III | 62 | ductal | Negative | Negative | Positive | 6743,10 | O+ | 97 |
| Fincyt-024-16 | II | 67 | lobular | Positive | Positive | Negative | 1381,70 | A+ | 96 |
| Fincyt-025-16 | I | 52 | ductal | Negative | Negative | Positive | 2277,50 | O+ | 94 |
| Fincyt-026-16 | II | 71 | ductal | Positive | Negative | Negative | 1482,30 | O+ | 86 |
| Fincyt-027-16 | III | 48 | ductal | Positive | Negative | Negative | 1851,30 | O+ | 81 |
| Fincyt-028-16 | II | 69 | lobular | Negative | Negative | Positive | 996,80 | B+ | 104 |
| Fincyt-029-16 | III | 45 | ductal | Negative | Negative | Negative | 1642,80 | O+ | 101 |
| Fincyt-030-17 | II | 51 | ductal | Positive | Negative | Negative | NA | NA | NA |
| Fincyt-031-17 | II | 49 | ductal | Positive | NA | NA | 2829,90 | A+ | 104 |
| Fincyt-032-17 | II | 38 | lobular | Positive | Positive | Negative | 2261,00 | B+ | 106 |
| INEN-01 | III | 48 | ductal | Positive | Negative | Negative | 1155,00 | O+ | 122,4 |
| INEN-02 | III | 48 | ductal | Negative | Negative | Positive | 648,00 | O+ | 99 |
| INEN-03 | III | 50 | ductal | Negative | Negative | Positive | 494,00 | O+ | 81 |
| INEN-04 | III | 59 | ductal | Negative | Negative | Negative | 331,00 | A+ | 97,2 |
| INEN-05 | III | 52 | other | Negative | Negative | Positive | 570,00 | O+ | 88,2 |
| INEN-06 | III | 57 | ductal | Positive | Positive | Positive | 541,00 | O+ | 88,2 |
| INEN-07 | IV | 50 | ductal | Positive | Negative | Negative | 558,00 | O+ | 82,8 |
| INEN-08 | II | 65 | mixed | Positive | Positive | Negative | 533,00 | O+ | 91,8 |
| INEN-09 | III | 32 | ductal | Positive | Positive | Negative | 450,00 | O+ | 84,6 |
| INEN-10 | III | 60 | ductal | Positive | Positive | Negative | 480,00 | O+ | 84,6 |
| INEN-11 | III | 61 | ductal | Positive | Positive | Negative | 555,00 | O+ | 88,2 |
| INEN-12 | III | 68 | ductal | Negative | Negative | Positive | 649,00 | O+ | 91,8 |
| INEN-14 | NA | NA | NA | NA | NA | NA | NA | NA | NA |
| INEN-15 | I | 53 | ductal | Negative | Negative | Negative | 1029,00 | O+ | 104,4 |
| INEN-16 | II | 77 | ductal | Positive | Negative | Negative | 472,00 | O+ | 91,8 |
| INEN-17 | IV | 59 | ductal | Positive | Positive | Positive | 653,00 | A+ | 93,6 |
| INEN-18 | II | 51 | ductal | Positive | Positive | Negative | 901,00 | O+ | 82,8 |
| INEN-19 | III | 51 | ductal | Negative | Negative | Positive | 728,00 | O+ | NA |
| INEN-20 | III | 57 | ductal | Negative | Negative | Negative | 655,00 | O+ | NA |
| INEN-21 | IV | 55 | ductal | Positive | Positive | Negative | 419,00 | NA | NA |
| INEN-22 | III | 50 | ductal | Negative | Negative | Negative | 673,00 | NA | 90 |
| INEN-23 | II | NA | ductal | Positive | Positive | Negative | 486,00 | O+ | 97,2 |
| INEN-24 | III | 64 | other | Positive | Negative | Negative | 581,00 | O+ | NA |
| INEN-25 | II | 59 | ductal | Negative | Negative | Positive | 1400,00 | O+ | 111,6 |
| INEN-26 | NA | 57 | ductal | Negative | Negative | Positive | 766,00 | O+ | 120,6 |
| INEN-27 | III | 79 | ductal | Positive | Positive | Negative | 570,00 | A+ | 104,4 |
| INEN-28 | II | 68 | ductal | Positive | Positive | Negative | 670,00 | O+ | 86,4 |
| INEN-29 | III | 42 | NA | Positive | Positive | Positive | 532,00 | A+ | NA |
| INEN-30 | III | 46 | ductal | Negative | Negative | Negative | 997,00 | O+ | 82,8 |
| INEN-31 | II | 51 | ductal | Negative | Negative | Negative | 408,00 | O+ | 79,2 |
| INEN-32 | III | 48 | ductal | Positive | Positive | Positive | 421,00 | A+ | 84,6 |
| INEN-33 | IV | 58 | ductal | Positive | Negative | Negative | 1092,00 | O+ | NA |
| INEN-34 | III | 47 | lobular | Positive | Positive | Positive | 630,00 | O+ | NA |
| INEN-35 | III | 58 | ductal | Positive | Positive | Negative | 479,00 | O+ | 82,8 |
| INEN-36 | II | 32 | ductal | Positive | Positive | Negative | 647,00 | O+ | 86,4 |
| INEN-37 | IV | NA | ductal | Negative | Negative | Negative | 499,00 | O+ | 86,4 |
| INEN-38 | NA | NA | NA | NA | NA | NA | NA | NA | NA |
| INEN-39 | III | 51 | ductal | Positive | Positive | Negative | 600,00 | O+ | 102,6 |
| INEN-40 | II | NA | ductal | Positive | Positive | NA | 714,00 | A+ | 91,8 |
| INEN-41 | III | 69 | ductal | Negative | Negative | Negative | 702,00 | O+ | 115,2 |
| INEN-42 | III | 71 | ductal | NA | NA | NA | 629,00 | A+ | 97,2 |
| INEN-43 | III | 32 | ductal | Negative | Negative | Negative | 573,00 | O+ | 90 |
| INEN-44 | III | 58 | ductal | Positive | Negative | Negative | 605,00 | O+ | 82,8 |
| INEN-45 | III | 48 | lobular | Positive | Positive | Negative | 468,00 | AB+ | 84,6 |
| INEN-46 | I | 45 | tubular | Positive | Positive | Negative | 340,00 | O+ | 82,8 |
| INEN-47 | III | 51 | ductal | Positive | Positive | Negative | 642,00 | NA | NA |
| INEN-48 | NA | NA | NA | NA | NA | NA | NA | NA | NA |
| INEN-49 | III | 51 | ductal | Positive | Negative | Negative | 588,00 | O+ | 115,2 |
| INEN-50 | III | 58 | ductal | Positive | Positive | Negative | 508,00 | O+ | 99 |
| INEN-64 | NA | NA | NA | NA | NA | NA | NA | NA | NA |
| INEN-65 | III | 36 | ductal | Negative | Negative | Positive | 546,00 | A+ | 81 |
| INEN-66 | NA | 21 | other | Negative | Positive | Negative | 697,00 | O+ | 90 |
| INEN-67 | III | 44 | lobular | Positive | Positive | Negative | 502,00 | O+ | 84,6 |
| Mean | | 53,88 |  | | | | 1121,49 |  | 93,23 |
| Standard Deviation | | 11,93 |  |  |  |  | 966,90 |  | 10,77 |

**
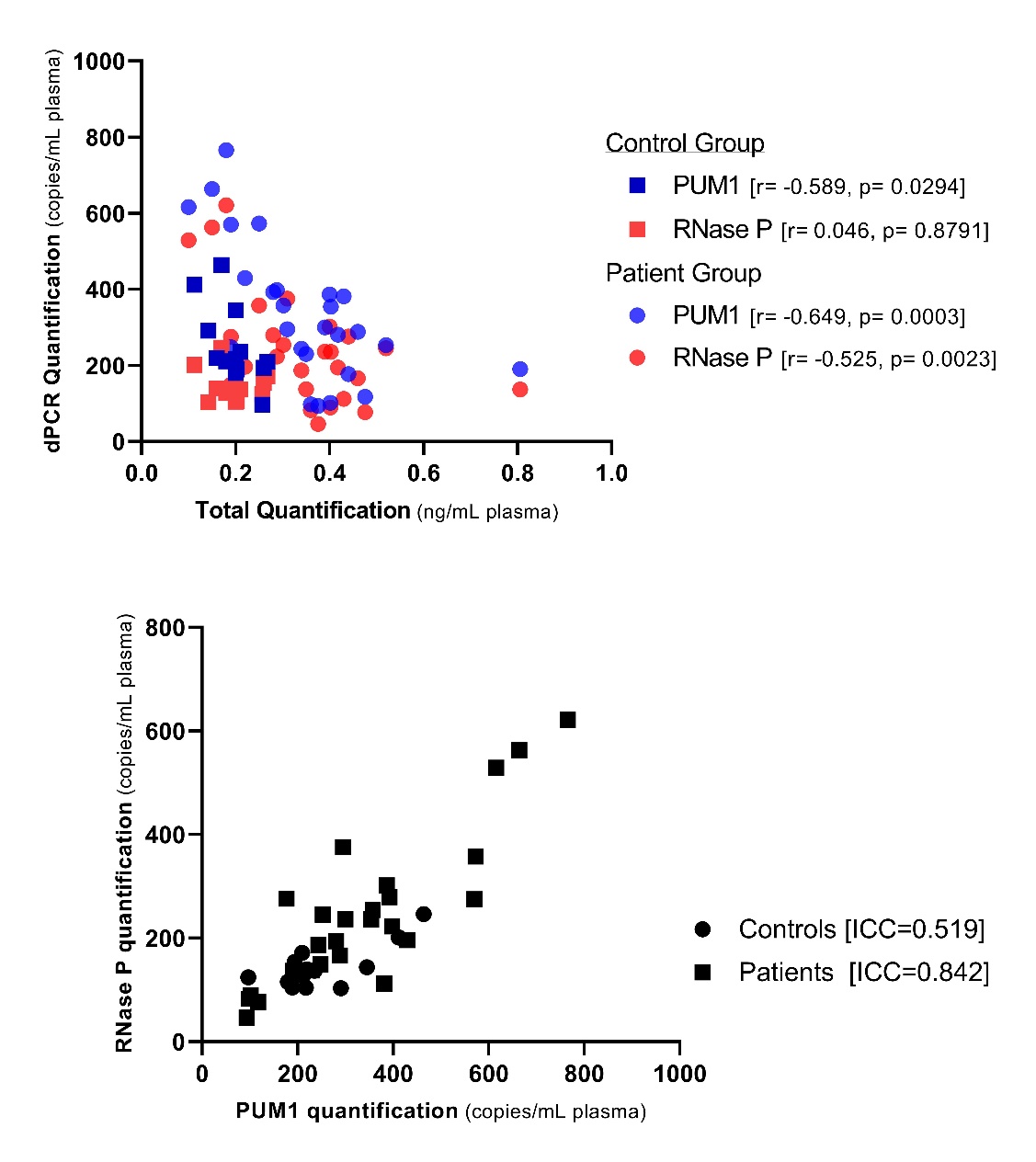
**

**Figure S1.** cfDNA does not represent a conserved genome. In a pilot study, we performed Qubit (nonspecific) and digital PCR (dPCR; targeting *PUM1* and RNase P) quantifications for a reduced group of samples. No correlation was found between Qubit and dPCR quantifications, which is evidence of the randomness of the chosen DNA fragments present in the plasma. *r* values: Spearman’s rank correlation coefficient; ICC: intraclass correlation coefficient.
